# Loss of CPEB3 Upregulates MEGF10 to Impair Mosaic Development of ON Starburst Amacrine Cells

**DOI:** 10.3389/fnmol.2016.00105

**Published:** 2016-10-24

**Authors:** Yin-Peng Chen, Geng-Shuo Bai, Meng-Fang Wu, Chuan-Chin Chiao, Yi-Shuian Huang

**Affiliations:** ^1^Institute of Biomedical Sciences, Academia SinicaTaipei, Taiwan; ^2^Institute of Neuroscience, National Yang-Ming UniversityTaipei, Taiwan; ^3^Institute of Systems Neuroscience and Department of Life Science, National Tsing-Hua UniversityHsinchu, Taiwan

**Keywords:** CPEB, MEGF10, retinal mosaic, starburst amacrine cells, translational control

## Abstract

Cytoplasmic polyadenylation element binding protein 3 (CPEB3) regulates target RNA translation in neurons. Here, we examined CPEB3 distribution and function in the mouse retina. CPEB3 is expressed in retinal neurons, including those located in the inner nuclear layer (INL) and ganglion cell layer (GCL) but not in cone and rod photoreceptors in the outer nuclear layer (ONL). A previous study found CPEB3 expressed in cholinergic starburst amacrine cells (SACs). We first examined these cells and observed aberrant SAC mosaicism in CPEB3-knockout (KO) retinas. Retinal neurons showed orderly spatial arrangements. Many individual subtypes are organized non-randomly in patterns called mosaics. Despite CPEB3 being expressed in both populations of SACs, OFF SACs in the INL and ON SACs in the GCL, aberrant mosaic regularity was observed in only ON SACs of CPEB3-KO retinas. Molecular characterization revealed that translation of multiple epidermal growth factor 10 (*Megf10*) RNA is suppressed by CPEB3 during the first week of postnatal development, when MEGF10 is primarily expressed in SACs and mediates homotypic repulsive interactions to define intercellular spacing of SACs. Thus, elevated MEGF10 expression in the absence of the translational repressor CPEB3 may account for the defective spatial organization of ON SACs. Our findings uncover for the first time that translational control plays a role in shaping retinal mosaic arrangement.

## Introduction

The cytoplasmic polyadenylation element binding (CPEB) family of RNA-binding proteins in vertebrates contains CPEB1, CPEB2, CPEB3 and CPEB4. They all share sequence similarity in their RNA-binding domains (Huang et al., 2006) and regulate target RNA translation in various tissues (see review in Ivshina et al., 2014). All CPEBs are present in the hippocampus (Wu et al., 1998; Theis et al., 2003; Huang et al., 2006; Chen and Huang, 2012), but mice with genetic ablation of *Cpeb1, Cpeb3* or *Cpeb4* exhibit different phenotypes in spatial memory (Berger-Sweeney et al., 2006; Chao et al., 2013; Tsai et al., 2013; Fioriti et al., 2015), so CPEBs have their own function *in vivo*.

CPEB3-regulated translation is important for synaptic plasticity and memory (Chao et al., 2013; Huang et al., 2014; Fioriti et al., 2015). Synaptic molecules such as the subunits of α-amino-3-hydroxy-5-methyl-4-isoxazolepropionic acid (AMPA) receptor and N-methyl-D-aspartate (NMDA) receptor and scaffolding postsynaptic density 95 (PSD95), are translationally regulated by CPEB3 in the hippocampal and cortical regions (Huang et al., 2006; Chao et al., 2013; Fioriti et al., 2015). Besides its function in the central nervous system, CPEB3 downregulates the translation of transient receptor potential vanilloid 1 (*Trpv1*) RNA in dorsal root ganglia to restrict the sensitivity of thermal nociception (Fong et al., 2016). Although CPEB3 RNA and protein are detected in some retinal neurons, including retinal ganglion cells (RGCs; Wang and Cooper, 2009), CPEB3-knockout (KO) mice recognized visual cues normally in spatial memory tasks (Chao et al., 2013). Because the accuracy of immunostaining depends on the antibody specificity, we used the CPEB3-KO retina as a negative control and an antibody generated in-house to re-evaluate the retinal expression pattern of CPEB3. Unexpectedly, we uncovered the role of CPEB3 in shaping retinal mosaic development.

Retinal neurons are organized in a layered structure. Within the layers, neurons of the same type are distributed in regular arrays (Scheibe et al., 1995; Galli-Resta et al., 1997, 1999). Such non-random architectures, termed retinal mosaics, are important to distribute each type of cell across the retina in an evenly spaced fashion to establish repeated arrangement of identical circuitry among different kinds of neurons. Such arrangement allows for parallel processing of visual stimuli from all receptive fields (Wassle and Riemann, 1978). Several mechanisms, including those regulating cell proliferation, fate determination, migration and apoptosis, contribute to the formation of retinal mosaics (see review in Reese, 2011). Once specific subtypes of neurons are in positions, other controls such as dendritic tiling and homotypic repulsion, may keep individual types of neurons in orderly planar arrays (Galli-Resta, 1998, 2000; Cook and Chalupa, 2000; Garrett and Burgess, 2011).

For example, in one of the most well-studied retinal mosaics, starburst amacrine cells (SACs) are randomly positioned in the outer neuroblast layer and then gradually migrate to the inner nuclear layer (INL) and ganglion cell layer (GCL) during early retinal development (Voigt, 1986; Millar et al., 1987). On postnatal day 1 (P1), SACs are orderly spaced and the mosaic pattern can be observed (Galli-Resta et al., 1997; Galli-Resta, 2000). Two transmembrane proteins, multiple epidermal growth factor-like domains 10 (MEGF10) and MEGF11, are specifically expressed in SACs and horizontal cells (HCs) during the critical period of mosaic development (i.e., from embryonic day 16 to the first postnatal week; Kay et al., 2012). Deletion of MEGF10 severely disrupts mosaic organization in SACs but not HCs unless in conjunction with MEGF11 deficiency. MEGF10 homotypic repellant signaling of neighboring MEGF10-expressing cells regulates mosaic spacing without affecting dendritic arborization of SACs (Kay et al., 2012). Thus, MEGF10 expression is critical to determine the repulsion force among SACs to define territorial organization.

SACs receive synaptic inputs from bipolar cells and make synaptic outputs to direction selective ganglion cells (DSGCs). Direction-selective responses to motion can be to the onset (ON) or offset (OFF) of illumination. Three DSGC and two SAC types respond to light (ON DSGCs and ON SACs), dark (OFF DSGCs and OFF SACs), or both light and dark moving objects (ON-OFF DSGCs; Vaney et al., 2012). ON and OFF SAC subpopulations have distinct locations of cell bodies in the GCL and INL, and their dendritic arbors co-stratify with the ON and OFF dendrites of the DSGCs, respectively (Famiglietti and Tumosa, 1987; Famiglietti, 1992; Hoshi et al., 2011). These connections establish the basic wiring of direction selectivity in the retina (Demb, 2007; Zhou and Lee, 2008; Lee et al., 2010; Yonehara et al., 2011). Ablation of SACs with an immunotoxin in the adult retina eliminates directional responses of DSGCs (Yoshida et al., 2001), which supports the critical role of SACs in motion-sensing vision.

In this study, we analyzed the retinal distribution of CPEB3 and mosaic spacing arrangement of SACs and HCs in CPEB3-wild-type (WT) and -KO mouse retinas. Deficiency of CPEB3 increased *Megf10* RNA translation and selectively affected the spacing arrangement of ON SAC mosaics.

## Materials and Methods

### Animals and Ethics Statement

This study was approved by the Institutional Animal Care and Use Committee (IACUC) of Academia Sinica and was compliant with the Taiwan Ministry of Science and Technology guidelines for ethical treatment of animals. All experimental protocols were performed in accordance with the guidelines of IACUC for the ethical treatment of animals and were made to minimize the number of mice used and their suffering. Mice were housed under a 12-h light/dark cycle in a climate-controlled room with *ad libitum* access to food and water. Appropriate anesthesia was applied for eyeball isolation as described below. Generation and characterization of CPEB3-KO mice in C57BL/6 genetic background were described before (Chao et al., 2013). CPEB3-WT and -KO mice were littermates from heterozygous mating. Both genders were used for this study.

### Antibodies

Antibodies used in the study were goat anti-choline acetyltransferase (ChAT, cat# AB144P) and rabbit anti-MEGF10 (cat# ABC10) from Millipore; rabbit anti-calbindin (cat# 300) from Swant; rabbit anti-glyceraldehyde 3-phosphate dehydrogenase (GAPDH, cat# SC25778) from Santa Cruz Biotechnology; mouse anti-CPEB3 and affinity-purified polyclonal rabbit anit-CPEB3 (homemade; Chao et al., 2012; Wang and Huang, 2012). Alexa Fluor-conjugated secondary antibodies were from Invitrogen.

### Retina Preparation and Immunofluorescence Staining

Animals were anesthetized with intraperitoneal injection of ketamine (10 mg/kg) and xylazine (10 mg/kg), and the eyeballs were enucleated with surgical scissors. After hemisection along the ora serrata, the lenses and vitreous humors were immediately removed. The posterior eyecups were then immersed in oxygenated (95% O_2_ and 5% CO_2_) artificial cerebrospinal fluid. For retinal slice preparation, the posterior eyecups were fixed with 4% formaldehyde for 30 min, transferred to 30% (wt/vol) sucrose in phosphate buffered saline (PBS) overnight at 4°C, then embedded in Tissue-Tek OCT compound. Retinas were sectioned vertically at 15 μm by use of a Leica cryostat. For whole-mount immunostaining, the retina was gently detached from the retinal pigment epithelium. Four radial cuts were made to facilitate flattening of the isolated retina, followed by 30 min fixation with 4% formaldehyde. Retinas were rinsed with PBS three times and incubated with primary antibodies in PBS containing 0.1% Trion X-100, 5% horse serum and 0.1% sodium azide for 5 days at 4°C with agitation. After a 1-h wash in six changes of PBS, secondary antibodies were applied overnight at 4°C with agitation. After a 1-h wash in six changes of PBS, retinas were flattened and mounted on slides with ganglion-cell side up. Similar staining conditions were applied to retinal slices, except the incubation time for primary and secondary antibodies was kept overnight and for 1 h, respectively.

### Imaging Acquisition and Quantification

All images (spatial resolution of 1024 × 1024) were acquired under a laser-scanning confocal microscope (LSM510 Meta, Zeiss) with a 20× air (Plan Apochromat, 0.75 NA, Zeiss) or 40× oil-immersion objective lens (Plan Apochromat, 1.3 NA, Zeiss). For imaging whole-mount stained retinas, we acquired confocal *z*-stacks obtained through the GCL and INL. To compare MEGF10 immunofluorescence signal between WT and KO samples, retinal slices obtained from four pairs of P5 WT and KO retinas were processed for immunostaining at the same time to minimize experimental variations. Images were acquired under the same exposure condition, which yielded the fluorescence intensity of ChAT and MEGF10 in the regions of interest (ROIs) with a given value ≤255 at each single pixel. The ROIs (i.e., soma and dendritic stratification of ON and OFF SACs) were selected manually and analyzed by using ImageJ v1.47. The mean fluorescence intensity of MEGF10 was quantified and normalized to that of ChAT by using ImageJ v1.47. In total, 24 images were analyzed for each genotype, with six images taken from each retinal slice. The total selected ROI areas in WT-ON SAC, KO-ON SAC, WT-OFF SAC and KO-OFF SAC are 67296, 65304, 54316 and 52774 μm^2^, respectively. To measure the diameter of SACs, these images were threshold adjusted to mark each cell, followed by particle analysis using ImageJ v1.47.

### Mosaic Regularity Analysis

We acquired confocal *z*-stacks through the GCL and INL from retinas stained with antibodies to ChAT and calbindin. Each retina was sampled in four locations: two in the middle and two in the peripheral area randomly selected from each of the four quadrants. Six retinas isolated from six WT or six KO mice at P5 or 2–3 months were used to generate 24 images per group for analyses. The center of each SAC or HC was marked manually to generate *X − Y* coordinates by using WinDRP software to compute density recovery profiles (DRPs; Rodieck, 1991) or by Ka-me software to analyze Voronoi domain areas (Khiripet et al., 2012). Four regularity measures were calculated for each image. The exclusion zone radius, the zone in which another cell is less likely to be found than would be expected for a random array, was computed from the DRP. The nearest neighbor (NN) distance for each cell was obtained. The collection of NN distances was then used to calculate the NN regularity index (NNRI), the mean NN distance divided by the standard deviation (SD; Raven et al., 2005a). The packing factor, a regularity index that ranges from 0 (a random array) to 1 (a perfect hexagonal array), was obtained from the DRP (Raven et al., 2005b). Another independent measurement, the Voronoi domain regularity index (VDRI), was calculated by dividing the mean Voronoi domain area by the SD. The Voronoi domain is a cellular territory area surrounded by neighbor cells (Raven et al., 2003; Keeley et al., 2007; Whitney et al., 2008). In the Ka-me software, a valid Voronoi domain is a polygonal cell area completely surrounded by other polygons, so cells at the border of images were not counted. Using this stringent criterion, smaller VDRIs were obtained but they were still larger than VDRIs calculated from random simulation (adult retina, real vs. simulation VDRI: 2.68 vs. 1.51 for WT-ON SAC; 2.35 vs. 1.46 for KO-ON SAC; 3.01 vs. 1.50 for WT-OFF SAC; 3.07 vs. 1.49 for KO-OFF SACs).

### Western Blot Analysis

Retinas were dissected from postnatal pups, homogenized in buffer (50 mM Tris-HCl, pH 7.5, 100 mM NaCl, 0.2% Triton X-100, 10% glycerol and 1× protease inhibitor cocktail [Roche]), and incubated on ice for 30 min. Homogenates were centrifuged at 13,000 rpm for 15 min at 4°C to remove cell debris. The supernatant was mixed with the sample buffer and incubated at 55°C for 20 min. Equal amounts of protein samples were separated on 10% sodium dodecyl sulfate polyacrylamide gel electrophoresis (SDS-PAGE) and then transferred to nitrocellulose membranes, which were blocked with 5% non-fat milk in TBST (10 mM Tris-HCl pH 8, 150 mM NaCl, 0.05% Tween 20) for 30 min, then incubated with the designated antibodies at 4°C overnight, followed by the corresponding horseradish peroxidase (HRP)-conjugated secondary antibodies. Signals were developed by using Immobilon Western chemiluminescent HRP substrate (Millipore).

### RNA Immunoprecipitation (RIP) Assay

Six retinas were lysed in 500 μl RIP buffer (20 mM HEPES, pH 7.4, 150 mM NaCl, 0.5% Triton X-100, 10% glycerol, 0.5 mM DTT, 1*X* protease inhibitor cocktail, and 40 U/ml RNase inhibitor), irradiated with 1500 Joules of UV (254 nm) light for 2 min on ice, then centrifuged at 12,000 rpm for 5 min. The resulting supernatant was divided equally and incubated with control or CPEB3 IgG-bound protein G beads at 4°C for 4 h. The beads were washed five times with 400 μl RIP buffer. One fifth of the beads eluted with Laemmli sample buffer was used for western blot analysis and the remaining beads were suspended in 400 μl RIP buffer containing 100 μg/ml proteinase K and 0.2% SDS for 10 min at 37°C. The supernatants were extracted with phenol/chloroform and precipitated with ethanol with 1 μg glycogen carrier to obtain RNA.

### RNA Extraction, CDNA Synthesis and RT-qPCR

Total RNA from retinas and cells was extracted with Omiczol reagent (Omics Bio, Taiwan). The cDNA was synthesized by using random primers and ImProm-II reverse transcriptase (Promega). Quantitative PCR (qPCR) involved the Universal Probe Library and LightCycler 480 system (Roche) and data analysis the comparative CT (threshold cycle value) method with non-CPEB3-targeted RNA and *Gapdh* as the reference. The PCR primers used were for *Megf10*, 5′-TCCCCATATGCAGAGATCAAC and 5′-TACTCCTTGCACAACGCTCA; *Pttg1*, 5′-GCCCTCTGAAGACACCCTTT and 5′-GCAATTCAACATCCAGAGTGG*; Barhl2*, 5′-CCAGAGCGACATCAAATGC and 5′-GGGACTCTCTCGGCTACTTG;* Cpeb3*, 5′-TCAACACAACGACATTGACAAA and 5′-CCCTGACACTCGTCACACAT; *Gapdh*, 5′-GCCAAAAGGGTCATCATCTC and 5′-CACACCCATCACAAACATGG; *Firefly luciferase*, 5′-TGAGTACTTCGAAATGTCCGTTC-3′ and 5′-GTATTCAGCCCATATCGTTTCAT-3′; *Renilla luciferase*, 5′- GGAGAATAACTTCTTCGTGGAAAC-3′ and 5′-GCTGCAAATTCTTCTGGTTCTAA-3′.

### Cell Culture, Transfection and Puromycin Selection

HEK-293T cells and Neuro-2a cells were cultured in Dulbecco’s modified Eagle’s medium (DMEM) supplemented with 10% fetal bovine serum. Neuro-2a cells were co-transfected with plasmids encoding myc-tagged CPEB3 (myc-CP3) and puromycin *N*-acetyl-transferase by using Lipofectamine 2000 following the manufacturer’s protocol. After overnight transfection, medium was replaced with fresh medium containing 5 μg/ml puromycin to eliminate untransfected cells for 48 h before harvesting cell lysates for immunoblotting.

### Plasmid Construction and Luciferase Reporter Assay

Mouse *Megf10* 3′-untranslated region (UTR) was PCR-amplified from retinal cDNA with the primers, 5′-ccgatatccaCATCAAAGGACTACTGGGT-3′ and 5′-ccgctcgagGGAAAGTTTATTGCATTCATC-3′. The DNA fragment was cloned into the pcDNA3.1-FLuc plasmid or pBluescript SK plasmid. HEK-293T cells were subcultured in a 12-well plate the day before transfection with the DNA mixture containing 20 ng plasmid expressing firefly luciferase reporter appended with mouse *Megf10* 3′-UTR, 40 ng plasmid expressing *Renilla* luciferase and 300, 600 or 750 ng plasmid expressing myc tag or myc-CPEB3, by using Lipofectamine 2000 (Invitrogen). The cells were harvested the next day for dual-luciferase reporter assay (Promega) following the manufacture’s protocol or RNA quantification by RT-qPCR.

### UV-Crosslinkig RNA Binding Assay

The recombinant MBP-CPEB3C fusion protein was purified as described (Huang et al., 2006). RNA probes for RNA binding assays were labeled by *in vitro* transcription with T3 or T7 RNA polymerase and α^32^P-UTP. For crosslinking, 20-μl reactions containing 3 × 10^5^ cpm labeled RNA, 20 μg heparin, 1 μg recombinant protein and yeast tRNA in 10 mM HEPES, pH 7.4, 50 mM KCl, 1 mM MgCl_2_, 0.5 mM DTT and 10% glycerol were kept on ice for 10 min, then irradiated with 1200 Joules of UV (254 nm) light for 5 min. UV-crosslinked samples were treated with RNase A and resolved on SDS-PAGE.

### Statistical Analysis

The sample size for both SACs and HCs included 24 images taken from six retinas isolated from adult or P5 mice of both genders. For analysis of cell density, exclusion zone, packing factor and VDRI, genotypes were compared by two-tailed Student’s *t* test; other tests were compared by two-tailed Student’s *t* test and one-way ANOVA. *P* < 0.05 was considered statistically significant.

## Results

### Broad Expression of CPEB3 in The INL and GCL of Adult Retinas

**Figure 1:**
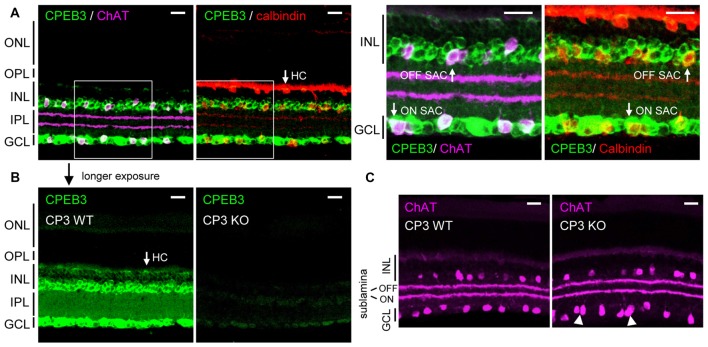
**Cytoplasmic polyadenylation element binding protein 3 (CPEB3) is expressed in neurons located in the inner nuclear layer (INL) and ganglion cell layer (GCL) of the retina. (A)** Retinal cryosections from adult mice were immunostained with CPEB3, choline acetyltransferase (ChAT) and calbindin antibodies. ChAT signal detected by the AlexaFluor 647-conjugated secondary antibody is pseudo-colored in magenta and used as a cholinergic starburst amacrine cells (SACs) marker. Calbindin-staining denoted both SACs and horizontal cells (HCs). **(B)** CPEB3 (CP3) and **(C)** ChAT immunostaining performed side-by-side in CP3-wild-type (WT) and -knockout (KO) retinas. Images with a saturated exposure condition from CP3-WT and -KO retinal slices show CPEB3-specific signals in the inner plexiform layer (IPL). Irregular spacing of ON SACs is denoted by arrowheads. ONL, outer nuclear layer; OPL, outer plexiform layer; Scales, 50 μm.

Previous *in situ* hybridization and immunostaining characterization revealed CPEB3 mRNA and protein widely distributed in the INL and GCL of adult retinas (Wang and Cooper, 2009). Nevertheless, CPEB3-KO mice do not have obvious visual impairment because they recognized visible cues in the Morris water maze task (Chao et al., 2013). To re-evaluate the retinal CPEB3 expression, we used affinity-purified CPEB3 polyclonal antibody for immunofluorescence staining of retina sections from 2-month-old CPEB3-WT and -KO mice. Retinal neurons are organized in five alternating stacked layers between three cell body layers and two neurite layers. The cell bodies are located in the outer nuclear layer (ONL), INL and GCL, and the processes are located in the outer plexiform layer (OPL) and inner plexiform layer (IPL). Similar to the reported expression pattern (Wang and Cooper, 2009), CPEB3 protein was widely detected in neurons in both the INL and GCL (Figure 1). The INL contains bipolar cells, HCs and OFF SACs among other types of amacrine cells, whereas the GCL contains ganglion neurons and ON SACs among other types of amacrine cells. Use of antibodies for ChAT to label cholinergic SACs and calbindin to label SACs and HCs revealed CPEB3 expressed in both cell types (Figure 1A), at a higher level in SACs than HCs (Figure 1B). In contrast to the previous finding of strong CPEB3-immunostaining signal in IPL neurites (Wang and Cooper, 2009), most of the CPEB3 was concentrated in somata instead of processes (Figure 1B). The dendrites of OFF and ON SACs stratify in the adjacent OFF and ON sublaminae of the IPL (Hayden et al., 1980). The two dendritic stratifications were outlined by immunostaining for ChAT (Figure 1C) but not CPEB3 (Figure 1B). CPEB3 immunoreactivity is specific because it was almost undetectable in the CPEB3-KO retina even under longer exposure condition (Figure 1B). Of note, CPEB3-KO retinal sections showed irregular spacing of ChAT-positive SACs in the GCL (i.e., ON SACs) but not the INL (i.e., OFF SACs; Figure 1C, arrowheads).

### Irregular ON SAC Mosaic Spacing in CPEB3-KO Adult Mouse Retina

**Figure 2:**
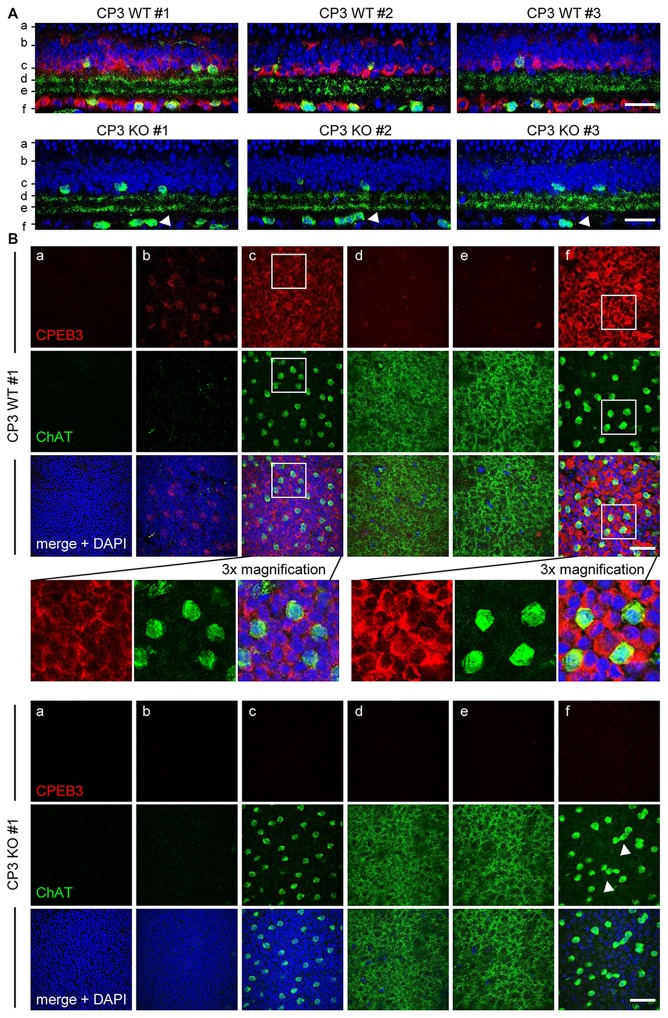
**Retinal layer organization appears normal in CPEB3-KO adult retina. (A)** Whole-mount retinas isolated from adult WT and KO mice (*n* = 3 per genotype) were immunostained with CPEB3 and ChAT and labeled with 4′,6-diamidino-2-phenylindole (DAPI). Confocal *z*-stacks obtained through the GCL and inner ONL were composited to show a *z*-axis cross section of each retina. **(B)** Cross-section images at the corresponding positions **(a–f)** in **(A)**. The magnified images from CP3WT#1 retina show several offset (OFF) SACs and onset (ON) SACs expressing CPEB3 and ChAT. Irregular spacing of ON SACs is denoted by arrowheads. **(a)** ONL; **(b,c)** outer and inner layers of INL; **(d,e)** OFF and ON sublamina; **(f)** GCL. Scales, 50 μm.

To ensure that the defect in spacing was not a sectioning artifact, CPEB3-WT and -KO whole-mount retinas were immunostained for ChAT and CPEB3. The *z*-axis cross sections of composite *z*-stack images show that the arrangement of ON but not OFF SACs appeared more disordered in the CPEB3-KO than -WT retinas (Figures 2A,B, arrowheads). Similarly, CPEB3 expression was abundant in the soma of both INL and GCL neurons including SACs (Figure 2B). Because of the thickness of retinas and the penetration efficiency of antibodies, CPEB3- and ChAT-immunostained signals were lower in OFF than ON SACs in whole-mount retinas. Moreover, dendritic arborizations in OFF and ON sublamina were comparable between WT and KO groups (Figure 2B,d,e sections). From the images of three pairs of WT and KO retinas, no overt changes in retinal thickness and gross organization of retinal layers were observed in the absence of CPEB3 (Figure 2A).

**Figure 3:**
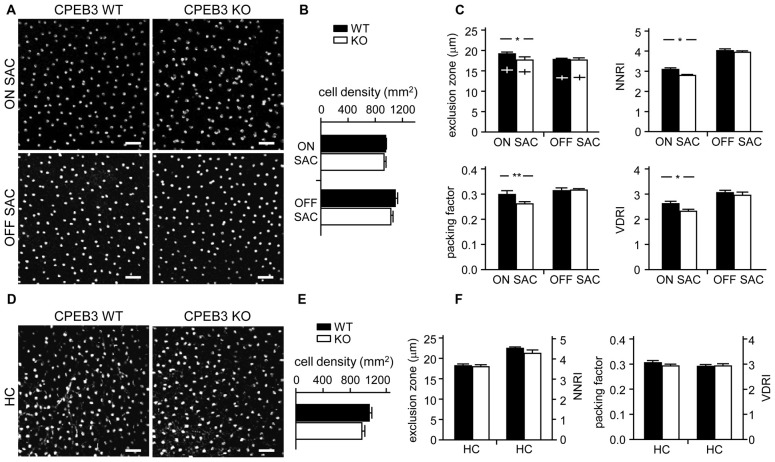
**Decreased spacing regularity in ON SAC mosaic of CPEB3-KO adult retina. (A)** Representative ON and OFF SAC images of whole-mount ChAT-immunostained CP3-WT and -KO adult retinas and **(B)** cell density. **(C)** Four variables used to calculate mosaic regularity of ON and OFF layers of SACs: exclusion zone, nearest neighbor regulatory index (NNRI), packing factor and Voronoi domain regularity index (VDRI). Cross signs (+) inside the exclusion zone bars represent the cell diameter. **(D)** Representative HC images of whole-mount calbindin-immunostained CP3-WT and -KO adult retinas and **(E)** cell density. **(F)** Mosaic regularity assessed by the same four variables. Scales, 50 μm. Data are mean ± SEM from 24 images taken from six retinas isolated from six adult mice per genotype. **P < 0.01, *P < 0.05 by Student’s *t* test.

To quantify the degree of spacing regularity, whole-mount retinas prepared from WT and KO adult mice were immunostained for ChAT (Figure 3A) and calbindin (Figure 3D). We examined four variables: exclusion zone, NNRI, packing factor and VDRI. Although the CPEB3-KO retinas showed normal cell density (Figure 3B) and cell diameter (Figure 3C) in both types of SACs, regularity was reduced in cell arrangements only in ON but not OFF SACs (Figure 3C). The exclusion zone parameter measures the mean diameter of exclusive area where no adjacent cells can be found. The NNRI is the mean distance to the closest neighboring cells divided by SD, so a disorganized cell array with higher SD results in lower NNRI. Both indices were significantly and specifically decreased for CPEB3-deficient ON SACs (Figure 3C). This finding was further supported by the analyses of packing factor and VDRI (Figure 3C). Similar to OFF SACs, in CPEB3-expressing HCs, the cell density and spacing regularity (Figures 3D–F) were comparable between CPEB3-WT and -KO groups.

### Disrupted Mosaic Arrangement of ON SACs in Developing CPEB3-KO Retina

**Figure 4:**
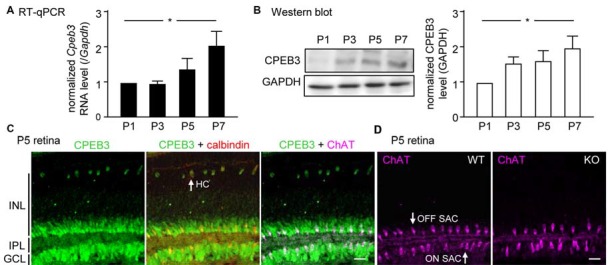
**Increasing expression of CPEB3 in the retina during the first postnatal week of development.** Retinas isolated from three mice at postnatal day (P) 1, P3, P5 and P7 were used for **(A)** RT-quantitative PCR (qPCR) to determine RNA levels of *Cpeb3* and *Gapdh* or **(B)** western blot analysis to measure the protein level of CPEB3 and GAPDH. Data are mean ± SEM RNA and protein levels of CPEB3 normalized to those of GAPDH. *P < 0.05 by one-way ANOVA. **(C)** Retinal cryosections from P5 pups immunostained with CPEB3, ChAT and calbindin antibodies. Calbindin staining denoted both SACs and HCs. ChAT signal was pseudo-colored in magenta to mark ON and OFF SACs.** (D)** ChAT immunostaining in P5 CP3-WT and -KO retina. Scales, 50 μm.

**Figure 5:**
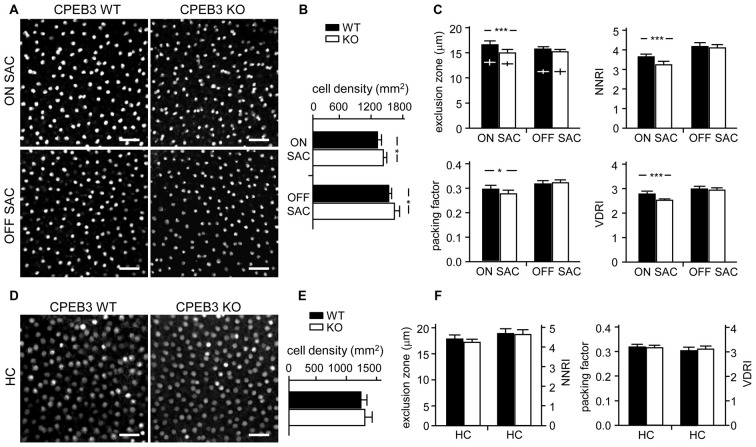
**Decreased spacing regularity in ON SAC mosaic of CPEB3-KO P5 retina. (A)** Representative ON and OFF SAC images of whole-mount ChAT-immunostained CP3-WT and -KO P5 retinas and **(B)** cell density. **(C)** Four variables used to calculate mosaic regularity of ON and OFF layers of SACs. Cross signs (+) inside the exclusion zone bars represent the cell diameter. **(D)** Representative HC images of whole-mount calbindin-immunostained CP3-WT and -KO P5 retinas and **(E)** cell density. **(F)** Mosaic regularity assessed with the same four variables. Scales, 100 μm. Data are mean ± SEM from 24 images taken from six retinas isolated from six P5 pups per genotype. ***P < 0.001, *P < 0.05 by Student’s *t* test.

Although CPEB3 is expressed in HCs and both SAC populations, the loss of CPEB3 perturbs mosaic formation within ON SACs alone. Thus, CPEB3 might regulate the synthesis of key molecules involved in SAC mosaic arrangement. Two transmembrane proteins, MEGF10 and MEGF11, are expressed in both SACs and HCs (Kay et al., 2012). MEGF10 expression is highest during the first postnatal week and decreases thereafter, and MEGF11 expression appears after the formation of SAC mosaics. Moreover, both SAC mosaics of MEGF10- but not MEGF11-KO retina showed severe disarrangement as early as in the first postnatal week (Kay et al., 2012). In contrast, ablation of the transcription factor *pituitary tumor-transforming gene 1* (*Pttg1*) in mice reduces the regularity of SACs only in adult but not P6 retinas, so PTTG1 maintains spacing regularity of the SAC mosaic after it is established (Keeley et al., 2014). In addition, the 3′-UTR of *Megf10* (3.4-kb) but not *Pttg1* (40-bp) RNA contains several potential CPEB3-binding sequences. Thus, if CPEB3 regulates translation of *Megf10* RNA to control SAC mosaic spacing, CPEB3 must be expressed in early postnatal retinas and a similar spacing defect would be recapitulated in developing CPEB3-KO retinas. Indeed, both CPEB3 RNA (Figure 4A) and protein (Figure 4B) expression was detected in the P1 retina and continued to increase to P7 (Figures 4A,B). Immunofluorescence staining of P5 retinas also showed wide distribution of CPEB3 in neurons of the INL and GCL (Figure 4C) and disorganized ON SAC array in the CPEB3-KO retina (Figure 4D). On quantifying whole-mount ChAT-stained P5 retinas (Figure 5A), the cell densities were slightly greater for both SAC types in the CPEB3-KO than -WT retinas (Figure 5B) but the cell diameters were comparable (Figure 5C). Spacing regularity was decreased in only the ON but not OFF layer of SACs in the P5 KO retinas (Figure 5C). CPEB3-WT and -KO groups showed no difference in cell density or spacing regularity in P5 HC mosaics (Figures 5D–F). Thus, ON SAC mosaic arrangement is regulated by CPEB3 during early postnatal development.

### CPEB3 Inhibits Translation of *MEGF10* RNA

**Figure 6:**
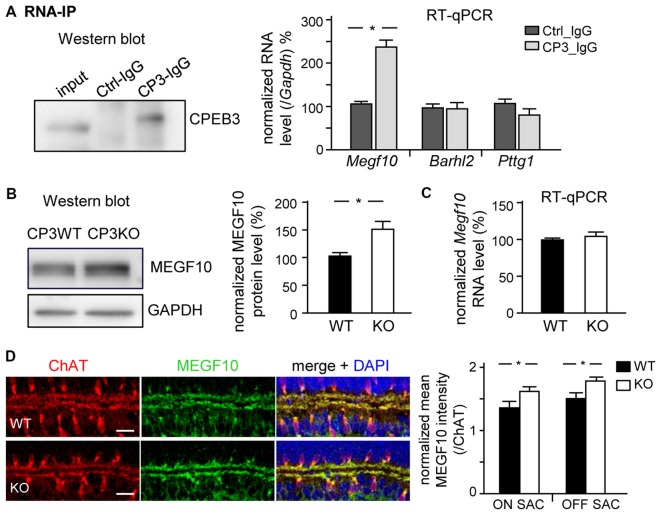
**CPEB3 deficiency increases the protein but not RNA level of multiple epidermal growth factor 10 (MEGF10) in P5 retina. (A)** RNA immunoprecipitation (RNA-IP). Retinal lysates from P5 neonates incubated with control (Ctrl) or CPEB3 (CP3) IgG. RT-qPCR analysis of *Megf10, Barh-like 2* (*Barhl2*) and* Pttg1* RNA levels relative to *Gapdh* RNA level. P5 CP3-WT and -KO retinas were used for **(B)** western blot analysis and **(C)** RT-qPCR expressed as relative ratios after normalization to GAPDH level. Data are mean ± SEM from three independent experiments. **(D)** P5 retinas isolated from CP3-WT and -KO pups (*n* = 4 for each group) were immunostained with ChAT and MEGF10 antibodies and labeled with DAPI. Representative images from CP3-WT and KO retinas are shown. Scales, 20 μm. Data are normalized mean ± SEM intensity of MEGF10 in ON and OFF SACs analyzed from 24 images taken from four retinas isolated from four pups per genotype. *P < 0.05 by Student’s *t* test.

**Figure 7:**
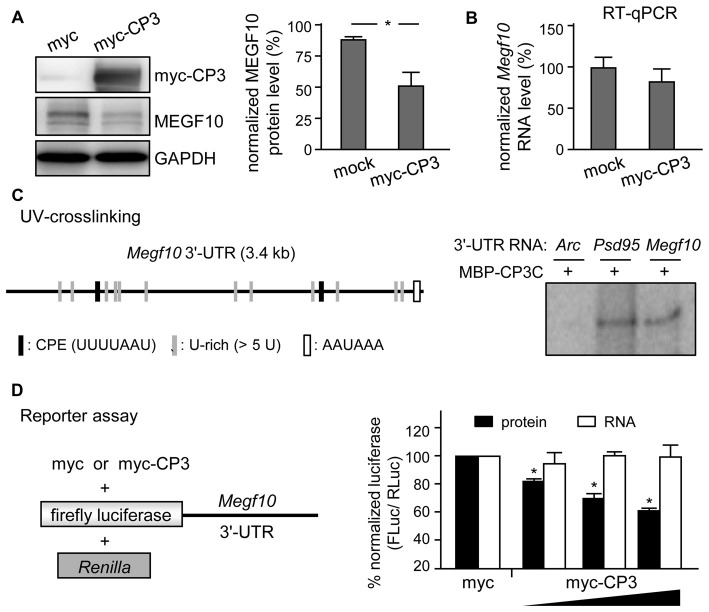
**CPEB3 represses translation of *Megf10* RNA via 3′-untranslated region (UTR). (A)** Neuro-2A cells co-transfected with plasmids expressing N-acetyl-transferase along with myc-tag or myc-tagged CPEB3 (myc-CP3) were under puromycin selection for 2 days to measure **(A)** MEGF10 protein level by immunoblotting and **(B)** *Megf10* RNA level by RT-qPCR. **(C)** CPEB3 bound to the *Megf10* 3′-UTR, which contains two consensus CPEs and multiple U-rich sequences besides the polyadenylation signal, AAUAAA. The recombinant maltose binding protein (MBP) fused to the C-terminus of CPEB3 RNA-binding domain (MBP-CP3C) was UV-crosslinked with ^32^P-labeled 3′-UTRs of *Arc* (a negative control) and postsynaptic density 95 (*Psd95*; a positive control) RNAs, treated with RNase, then analyzed by sodium dodecyl sulfate polyacrylamide gel electrophoresis (SDS-PAGE). **(D)** HEK-293T cells were transfected with the reporter plasmids, firefly luciferase (FLuc) appended with mouse *Megf10* 3′-UTR and *Renilla* luciferase (RLuc), along with the increasing amount of plasmid expressing myc-tag or myc-CP3. Normalized luciferase activity (FLuc/RLuc) was calculated with that in the myc control group arbitrarily set to 1. Data are mean ± SEM from three independent experiments. *P < 0.05 by Student’s *t* test.

MEGF10 expression in P5 retinas is restricted to SACs and HCs (Kay et al., 2012), so we used CPEB3-WT and -KO P5 retinas to examine whether CPEB3 affects the translation of *Megf10* RNA. We first immunoprecipitated CPEB3 from P5 retinal lysates and analyzed the amount of co-precipitated RNAs, including *Megf10, Barh-like 2 (Barhl2), Pttg1 and Gapdh*. Deletion of the transcription factor *Barhl2* in mice reduces the number of RGCs and alters the composition of amacrine cell subtypes, thereby resulting in a two- to three-fold increase in both SAC populations in *Barhl2*-null P21 retinas (Ding et al., 2009). Because the cell densities of ON and OFF SACs were slightly elevated in CPEB3-KO P5 retinas (Figure 5B), we also examined whether CPEB3 could bind to *Barhl2* RNA *in vivo*. IgG-precipitated RNAs were a control for non-specific RNA binding. *Megf10* but not *Barhl2, Pttg1 and Gapdh* RNA was enriched in CPEB3 immunoprecipitates (Figure 6A). Moreover, MEGF10 protein level (Figure 6B) was upregulated about 50% but not RNA level (Figure 6C) in the P5 CPEB3-KO retinas. Immunohistochemistry also revealed an approximately 18% increase in MEGF10 expression in both ON and OFF SACs (Figure 6D), so inhibited MEGF10 synthesis is not ON SAC-specific. Because MEGF10 is mostly detected in SACs, with a very mild signal present in HCs of P5 retinas, the western blot analysis should give more accurate assessment than immunostaining in determining the change in MEGF10 expression. To further support that CPEB3 functions as a translational repressor to confine MEGF10 expression, we used Neuro-2a cells, which express endogenous MEGF10 (Singh et al., 2010) and a very low amount of CPEB3 (Figure 7A). Ectopic expression of myc-CP3 downregulated level of MEGF10 protein (Figure 7A) but not RNA (Figure 7B). The 3′-UTR of *Megf10* RNA contains several potential CPEB3-binding sequences (Pavlopoulos et al., 2011; Chao et al., 2013), including two consensus CPEs (UUUUAAU) and multiple U-rich sequences (Figure 7C). To determine whether CPEB3 directly binds to *Megf10* RNA, the radiolabeled 3′-UTRs of *Arc* (a negative control), *Psd95* (a positive control; Chao et al., 2013) and *Megf10* RNAs were subjected to *in vitro* UV crosslinking with the C-terminal RNA-binding domain of CPEB3 fused to maltose-binding protein (MBP-CPEB3C). The *in vitro* binding assays demonstrated that CPEB3 interacted with *Megf10* RNA (Figure 7C). Additionally, reporter assay with firefly luciferase (FLuc) appended to the *Megf10* 3′-UTR showed increasingly inhibited synthesis of FLuc protein, but not RNA, accompanied by increased myc-CP3 expression (Figure 7D). Thus, CPEB3 binds to the *Megf10* 3′-UTR and downregulates *Megf10* translation.

## Discussion

This study demonstrates that CPEB3-controlled translation regulates retinal mosaic development. Deletion of CPEB3 increased the expression of MEGF10, an important molecule to define proper spacing among neighboring SACs via homotypic repulsion. MEGF10-KO mice show severely disorganized SAC mosaics and ectopic overexpression of MEGF10 in other retinal neurons repels adjacent SACs (Kay et al., 2012), so the level of MEGF10 must be tightly controlled through development. Here, we identified that CPEB3 plays a role in shaping the SAC mosaic arrangement by regulating the translation of *Megf10* RNA in developing retinas.

If MEGF10 regulates the mosaic formation of both SAC subpopulations, ON and OFF, we wondered why the moderate increase in MEGF10 expression (Figure 6D) in CPEB3-KO retinas selectively affected the organization of ON but not OFF SACs (Figures 3, 5). SAC mosaics develop during late embryonic stages once the newborn SACs in the outer neuroblast layer migrate to their final laminar locations to form OFF and ON SACs in the INL and GCL, respectively (Voigt, 1986; Millar et al., 1987). Notably, during early postnatal development, the cell density in both ON and OFF SACs is slightly elevated in P5 CPEB3-KO retinas (Figure 5B). Because the number of SACs remains unchanged in MEGF10-null retinas (Kay et al., 2012), CPEB3 should affect expression of other molecules, which are likely involved in proliferation, apoptosis and/or fate specification of SACs, to cause such a transient increase during retinal development. The unidentified factors and whether the slightly increased SAC population contributes to spacing irregularity of ON SACs require further investigation. Despite high CPEB3 level in RGCs of the GCL, RGCs do not likely affect the spacing arrangement of ON SACs because experimental alteration of the RGC number via optic nerve lesion or overexpression of anti-apoptotic *Bcl2* does not affect SAC organization (Galli-Resta, 2000). Dendritic arborizations of MEGF10-KO SACs develop normally to overlap with those of their neighboring cells, so MEGF10-mediated homotypic repulsive interaction among SACs instead of dendritic tiling is critical for SAC mosaic formation (Kay et al., 2012). Indeed, depletion of PlexA2, specifically expressed in SACs, disrupts two sublaminar dendritic stratifications in IPL without affecting ON and OFF SAC mosaic regularity (Sun et al., 2013). In agreement with these findings, we also observed aberrant mosaic arrangement but not dendritic stratification of ON SACs in CPEB3-null retinas (Figures 1C, 2).

Because homotypic repulsive interaction is the key factor determining the cellular spacing arrangement of SACs (Kay et al., 2012; Reese, 2012; Sun et al., 2013), a moderate increase in MEGF10 molecules evenly distributed on the surface of cells should generate more repellant force between two adjacent SACs in a short rather than long distance. Moreover, such an increased force pushing evenly in all directions should nullify its effect in a perfect symmetric array but have more impact on an array with low regularity. Previous studies found the spatial organization of ON SACs less regular than OFF SACs across multiple mouse strains including C57BL/6 (Galli-Resta, 2000; Whitney et al., 2008). This discrepancy was suggested to be due to ON SACs in the GCL being passively displaced by bundles of optic axons and vasculature expansion during development. Cell number, packing factor, NNRI and VDRI were lower in ON SAC mosaics in a previous study (Whitney et al., 2008) and in our P5 and adult CPEB3-WT retinas (Figures 3, 5). From regularity index analysis, OFF SACs appear to form a more regular array than ON SACs. Thus, elevated MEGF10-mediated repulsion force should have more significant impact in an array with low than high regularity, for selective disruption of ON but not OFF SACs in CPEB3-KO retinas. The MEGF10-heterozygote (MEGF10^+/−^) also affects the mosaic arrangement of OFF SACs, but the defect is much less severe than with MEGF10-KO (Kay et al., 2012). So far, no detailed analyses about the spacing regularity of ON SACs in MEGF10^+/−^ and MEGF10-KO have been published. If our hypothesis is correct, MEGF10 haploinsufficiency may differentially affect spacing regularity in ON and OFF SACs. Although the *Barhl2* and *Pttg1* RNA levels remain unchanged in CPEB3-KO retinas (data not shown) and CPEB3 does not bind to both RNAs *in vivo* (Figure 6A), we cannot exclude the possibility that other unidentified molecules or compensatory mechanisms may also differentially contribute to shaping ON and OFF SAC mosaicism under CPEB3 deficiency.

Although the previous study demonstrated that MEGF10-mediated homotypic interaction causes intercellular repulsion, how MEGF10 produces repulsive signals remains undetermined at the molecular level. Kay et al. (2012) were unable to demonstrate homophilic binding of MEGF10 molecules by biochemical methods. MEGF10 is a single-spanning transmembrane protein with an EMI domain followed by 17 epidermal growth factor-like domains at the extracellular N-terminus. The intracellular C-terminus contains phosphotyrosine-binding and Src homology 2 domains, which are important motifs to initiate signal transduction. Ectopic overexpression of the WT and also the intracellular domain-deleted mutant MEGF10 in retinas is sufficient to repel adjacent SACs (Kay et al., 2012). In contrast, overexpression of only the WT but not mutant MEGF10 in adjacent HEK293 cells forms a jigsaw puzzle-like pattern with a sharp boundary gap <2 μm (Kay et al., 2012). Moreover, we found Neuro-2A cells with or without CPEB3 overexpression to downregulate MEGF10 did not form a boundary gap (data not shown). Thus, MEGF10-mediated repulsive signaling to define cellular territory may vary substantially in different cells and require further investigation to clarify its mechanistic details.

Local protein synthesis in response to chemotropic cues is one mechanism to steer growth cone navigation to corresponding targets in the developing nervous system (Erskine and Herrera, 2007; Shigeoka et al., 2013). RGCs cultured from *Xenopus* tectum is an *in vitro* model system to dissect signaling pathways and molecular components involved in growth cone turning and pathfinding. Although CPE (i.e., UUUUAAU or similar sequence)-mediated translation regulates chemotropic responses in axonal growth cones of *Xenopus* RGCs *in vitro* and *in vivo*, blocking CPEB1 expression with morpholino oligonucleotides was not sufficient to impair retinal axon guidance (Lin et al., 2009), so other CPE-binding proteins such as CPEBs2-4 may redundantly control such responses (Wu et al., 1997; Lin et al., 2009). Another study identified impaired visual avoidance behavior in *Xenopus* tadpoles (i.e., to escape an approaching object by changing a swim trajectory) by inhibiting CPEB1 synthesis with morpholinos (Shen et al., 2014). Nevertheless, such a CPEB1-dependent retinotectal circuit plasticity induced by visual conditioning was demonstrated in only postsynaptic optic tectal neurons but not RGCs (Shen et al., 2014). Although CPEB1-, CPEB3- and CPEB4-KO mice have a normal ability to recognize visual cues in the Morris water maze test (Berger-Sweeney et al., 2006; Chao et al., 2013; Tsai et al., 2013), whether they have other retinal defects has not been closely examined. In our experience, the loss of CPEB3-controlled translation affects only its target expression by less than 1.5 to 2-fold. Such a mild change is sufficient to cause abnormalities in memory and thermosensation (Chao et al., 2013; Fong et al., 2016), as does ON SAC mosaic regularity. Our study identifies the role of CPEB3 in controlling ON SAC mosaic development. Further investigation is needed to determine whether a moderate disorganization in ON SAC mosaics can affect direction-selective responses of ON- and ON-OFF DSGCs and motion-sensing vision in CPEB3-KO mice.

## Author Contributions

Y-PC, G-SB and M-FW performed the experiments and analyzed the data. Y-SH supervised the study with the guidance from C-CC and wrote the manuscript with contributions from Y-PC and G-SB.

## Funding

This work was supported by the Taiwan Ministry of Science and Technology (MOST 102-2628-B-001-007-MY3, MOST104-2321-B-001-064) and Academia Sinica (AS-103-TP-B05).

## Conflict of Interest Statement

The authors declare that the research was conducted in the absence of any commercial or financial relationships that could be construed as a potential conflict of interest.
